# Use and Influencing Factors of mHealth Services Among Adult Survivors of Cancer: Cross-Sectional Survey Study

**DOI:** 10.2196/82902

**Published:** 2026-04-23

**Authors:** Taiguo Liu, Lina Duo, Musi Zhang, Jianjun Zhang, Jingna Zhang, Xiao Sun, Haoyue Deng, Ji Cheng, Ya Sun

**Affiliations:** 1 Department of Oncology Chengdu Seventh People’s Hospital (Affiliated Cancer Hospital of Chengdu Medical College) Chengdu China; 2 Department of Dermatology & Medical Aesthetics Chengdu Seventh People’s Hospital (Affiliated Cancer Hospital of Chengdu Medical College) Chengdu China; 3 Department of Radiation Oncology Sichuan Cancer Hospital&Institute, Sichuan Cancer Center, University of Electronic Science and Technology of China Chengdu China; 4 Department of Gynecology and Obstetrics West China Second Hospital, Sichuan University/Key Laboratory of Birth Defects and Related Diseases of Women and Children (Sichuan University), Ministry of Education Chengdu China; 5 Department of Palliative Medicine West China Fourth Hospital, Sichuan University Chengdu China

**Keywords:** survivor of cancer, mobile health, mHealth, adoption, influencing factor, China, cross-sectional study

## Abstract

**Background:**

The growing number of survivors of cancer in China has created an increasing need for survivorship care as many survivors face ongoing physical, psychological, and social challenges after treatment. Mobile health (mHealth) services, which are delivered through mobile devices and apps, have emerged as potential tools to support self-management, facilitate access to care, and improve quality of life. However, evidence on the prevalence, use patterns, and determinants of mHealth adoption among Chinese survivors of cancer remains limited.

**Objective:**

This study aimed to examine the prevalence and patterns of mHealth use among adult survivors of cancer in China and identify sociodemographic and clinical factors associated with adoption.

**Methods:**

We conducted a multicenter cross-sectional survey study between February 13, 2024, and September 21, 2024, at 4 tertiary cancer care centers in China. Adult survivors of cancer were recruited using convenience sampling. Data were collected through structured face-to-face questionnaires assessing sociodemographic and clinical characteristics, mHealth use, perceived needs, concerns, and user experience. Missing data were limited and handled using complete-case analysis after the Little test supported consistency with missing completely at random. Descriptive analyses summarized participant characteristics and mHealth-related variables. Group differences were examined using chi-square tests and 2-tailed independent-sample *t* tests. Significant variables in univariate analyses were entered into a multivariable logistic regression model.

**Results:**

Of 1152 participants, 364 (31.6%) reported prior mHealth use. Use was concentrated in practical functions, particularly appointment booking (301/364, 82.7%), online consultation (244/364, 67%), and viewing examination or laboratory reports (215/364, 59.1%), with WeChat-based platforms being the most commonly used access channel (244/364, 67%). Participants reported high demand for clinical guidance (917/1152, 79.6%) and direct communication with health care professionals (901/1152, 78.2%), whereas common concerns included leakage of private information (694/1152, 60.2%) and inaccurate illness judgment (633/1152, 54.9%). In multivariable analysis, mHealth use was significantly associated with younger age, higher educational level, annual household income of at least ¥100,000 (US $14,527.90), widowed or divorced marital status, living alone, and treatment dissatisfaction; cancer type and time since diagnosis were not significant predictors.

**Conclusions:**

mHealth use among adult survivors of cancer in China is established but uneven, with use concentrated in practical service functions rather than comprehensive survivorship support. Uptake was significantly associated with age, socioeconomic position, social circumstances, and treatment experience but not with clinical characteristics. Survivors reported a strong demand for clinically relevant and communication-oriented functions but also expressed substantial concerns about privacy, accuracy, reimbursement, and physician authenticity. Future survivorship mHealth services should prioritize clinical relevance, trust, integration with formal care, and equitable implementation to achieve broader and more meaningful use.

## Introduction

The growing population of survivors of cancer has made survivorship care an increasingly important public health priority worldwide, with the number of adult survivors of cancer rising rapidly due to advances in early detection, treatment, and supportive care. In China, survivorship care is shaped by a combination of rapid growth in the survivor population, persistent urban-rural disparities in health care access and use, variable continuity of follow-up care, and uneven distribution of digital and supportive care resources, all of which may influence how survivors manage long-term physical, psychological, and social consequences after treatment [1,2]. Mobile health (mHealth) services, defined as the use of mobile devices and apps to deliver health information and support, have emerged as promising tools to address the complex needs of survivors of cancer by facilitating self-management, improving access to care, and enhancing quality of life (QOL) [3-5].

The literature on mHealth interventions for survivors of cancer is extensive and rapidly evolving. Systematic reviews and meta-analyses have consistently demonstrated that mHealth interventions can improve health-related QOL, self-efficacy, and psychological outcomes such as anxiety and depression among adult survivors of cancer [3,6-8]. For example, a recent meta-analysis of randomized controlled trials found that mobile app–based interventions significantly improved QOL and self-efficacy and reduced anxiety, depression, and distress, with short-term and interactive interventions showing the greatest benefits [3]. Similarly, mHealth interventions targeting physical activity and dietary behaviors have shown promise in promoting healthy lifestyles and improving behavioral outcomes among survivors of cancer, although most studies have focused on short-term effects and predominantly included college-educated participants [9-12]. The use of mHealth for symptom monitoring and management has also been shown to be feasible and acceptable, with high adherence rates and positive associations between reduced symptom burden and improved health-related QOL [13-15].

Despite encouraging findings, important gaps remain. Engagement and sustained use of mHealth tools are often suboptimal. Uptake and adherence may be influenced by technical barriers, competing priorities, age, digital literacy, and psychological status [7,16,17]. Rural-urban disparities have also been documented, with rural survivors of cancer being less likely than urban survivors to own mobile devices and use mHealth apps [18-20]. In China, studies have highlighted suboptimal health care use among survivors of cancer, particularly in rural areas, and identified socioeconomic status, health insurance coverage, and clinical factors as key determinants of service use [1,21]. While mHealth has the potential to bridge some of the gaps, research on its adoption and influencing factors among Chinese survivors of cancer remains limited.

Furthermore, the quality and effectiveness of mHealth interventions are highly variable, with significant heterogeneity in intervention design, delivery methods, theoretical underpinnings, and outcome measures [17,22]. Reviews have called for more rigorous, theory-driven research to establish best practices for mHealth intervention development, implementation, and evaluation, as well as greater attention to issues of equity, usability, and patient-centeredness [17,22,23]. The digital divide persists, with older adults, ethnic minority groups, and those with lower socioeconomic status less likely to benefit from mHealth innovations [9,19,20]. In the Chinese context, the adoption and impact of mHealth services are further shaped by urban-rural differences in service availability, differences in insurance and reimbursement arrangements, the central role of widely used super app platforms such as WeChat, and variations in health and digital literacy across survivor groups [1,2,21].

Despite the proliferation of mHealth interventions worldwide, there is a notable research gap regarding the use patterns and determinants of mHealth service adoption among adult survivors of cancer in China. Most existing studies have been conducted in Western populations or have focused on specific cancer types, short-term outcomes, or feasibility rather than comprehensive assessments of use and influencing factors in diverse Chinese settings [9,21,23]. There is a critical need for empirical data on how adult survivors of cancer in China engage with mHealth services, what factors facilitate or hinder their use, and how the services can be optimized to address the unique needs of this population.

The aim of this study was to examine the prevalence, use patterns, and correlates of mHealth service use among adult survivors of cancer in China. Using a multicenter cross-sectional design, we sought to identify sociodemographic, clinical, and care-related factors associated with mHealth adoption, as well as survivors’ needs, concerns, and perceived barriers.

## Methods

### Study Design and Participants

An institution-based cross-sectional survey study was conducted at 4 tertiary cancer care centers in China between February 13, 2024, and September 21, 2024. Convenience sampling was used to recruit adult survivors of cancer attending outpatient services at the participating centers. Eligible individuals were aged 18 years or older, had a confirmed cancer diagnosis, demonstrated adequate communication ability, were able to accurately report their personal health information, and provided written informed consent. Individuals with cognitive impairment who were unable to understand the questionnaire after explanation by the investigators were excluded.

### Sample Size Estimation

The sample size was calculated using the following formula:

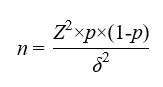



where *n* is the required sample size, *Z* is the standard normal deviate at a 95% confidence level (*Z=*1.96), *p* is the expected prevalence of mHealth use, and δ is the allowable error. On the basis of prior literature, the prevalence of mHealth use among survivors of cancer was assumed to be 30% (*P*=.30). The allowable error was set as a δ value of 0.1 and *p* value of 0.03 to ensure moderate precision. Thus, a minimum of 896 participants was required. To account for potential invalid responses, the sample size was inflated by 20%, resulting in a final target of approximately 1075 participants.

### Questionnaire Development and Validation

The questionnaire was developed based on prior literature on mHealth use among survivors of cancer and chronic disease populations [24-27] and was refined through consultation with 3 oncology specialists and 2 digital health researchers to ensure content validity. A pilot test was conducted with 30 adult survivors of cancer recruited from the Department of Oncology, Chengdu Seventh People’s Hospital, who were not included in the final analytic sample. The pilot confirmed face validity and comprehensibility and led to minor wording revisions. In the final sample, internal consistency was acceptable for the multi-item domains, with a Cronbach α of 0.842 for needs, 0.813 for concerns, and 0.764 for satisfaction. The final questionnaire contained 47 items. See Multimedia Appendix 1 for the questionnaire.

### Data Collection

Data were collected face-to-face using a structured questionnaire administered by trained health care professionals. All investigators underwent standardized training to ensure consistent questionnaire administration. Questionnaires did not include direct personal identifiers. Data were entered into a study dataset accessible only to the research team. To minimize duplicate participation, each participant completed the questionnaire once during on-site recruitment under investigator supervision.

### Questionnaire Measures

All study variables were collected through self-report using the structured questionnaire.

#### Sociodemographic and Clinical Characteristics

Information was collected on sex, age, educational level, annual household income, marital status, residence (urban or rural), occupation, cohabitation status, smoking and drinking habits, cancer type (solid vs nonsolid), time since diagnosis (<12 or >12 months), treatment modalities (surgery, chemotherapy, radiotherapy, targeted therapy, immunotherapy, or others), comorbid chronic conditions, and satisfaction with treatment.

#### mHealth Use

Participants were asked whether they had ever used mHealth services. For users, additional information was collected, including frequency of use, average duration per session, main platforms or apps used, functions accessed (such as online consultation, appointment booking, health information, or medication reminders), and satisfaction with the services.

#### Needs and Expectations for mHealth

Participants were asked about their perceived needs for various mHealth functions, including clinical guidance (such as medication, rehabilitation, and psychological support), communication with physicians, lifestyle guidance, home-based disease management, access to health information, regular follow-up, family assistance, peer support, electronic medical record access, and online purchasing of medicines or rehabilitation equipment.

#### Perceptions of Potential Problems

Concerns regarding mHealth use were assessed, including data inaccuracy, privacy risks, misjudgment of illness, reimbursement restrictions, authenticity of physician information (identity, credentials, and work experience), limitations in purchasing medicines, and service costs.

#### User Experience and System Performance Measures

Participants evaluated ease of use, system stability, prevalence of advertisements, concerns about fraud, operability of the system, ability to deliver disease-specific treatments, and integration with hospital information systems.

### Statistical Analysis

#### Overview

Data were entered into a database using Microsoft Excel and analyzed using SPSS software (version 26.0; IBM Corp). Descriptive statistics were used to summarize participant characteristics and mHealth-related variables, with continuous variables presented as means and SDs and categorical variables presented as frequencies and percentages. Group differences between mHealth users and nonusers were examined using chi-square tests for categorical variables and 2-tailed independent-sample *t* tests for continuous variables. Variables associated with mHealth use in univariate analyses at a *P* value of less than .05 were entered into the multivariable logistic regression model to identify factors independently associated with mHealth use. Adjusted odds ratios, 95% CIs, and *P* values are reported. A 2-sided *P* value of less than .05 was considered statistically significant.

#### Missing Data Handling

Missing data were assessed for all variables included in descriptive and regression analyses. The proportion of missingness for individual variables ranged from 0.3% to 4.6%, and the overall proportion of missing values across the analyzed variables was 2.1%. As the extent of missingness was low, we formally evaluated the missing data mechanism before selecting the analytic approach. The Little test suggested that the missing data pattern was consistent with missing completely at random (MCAR; *χ*^2^_47_=52.8; *P*=.26). On that basis, and because the proportion of missing data was small, we used complete-case analysis. No score prorating was performed.

### Ethical Considerations

The research protocol was reviewed and approved by the ethics committee of Chengdu Seventh People’s Hospital (approval KY0182). All participants provided written informed consent after being informed of the purpose, procedures, risks, and benefits of the study at the beginning of the survey. Participants’ privacy and confidentiality were protected by assigning unique study codes, storing data on encrypted servers with restricted access, and removing all personal identifiers prior to analysis. No identifiable participant information appears in the manuscript or supplementary files. Participants received no compensation. Data handling complied with local and international regulations on personal data protection, including the Helsinki Declaration.

## Results

### Participant Recruitment and Sample Characteristics

**Figure 1:**
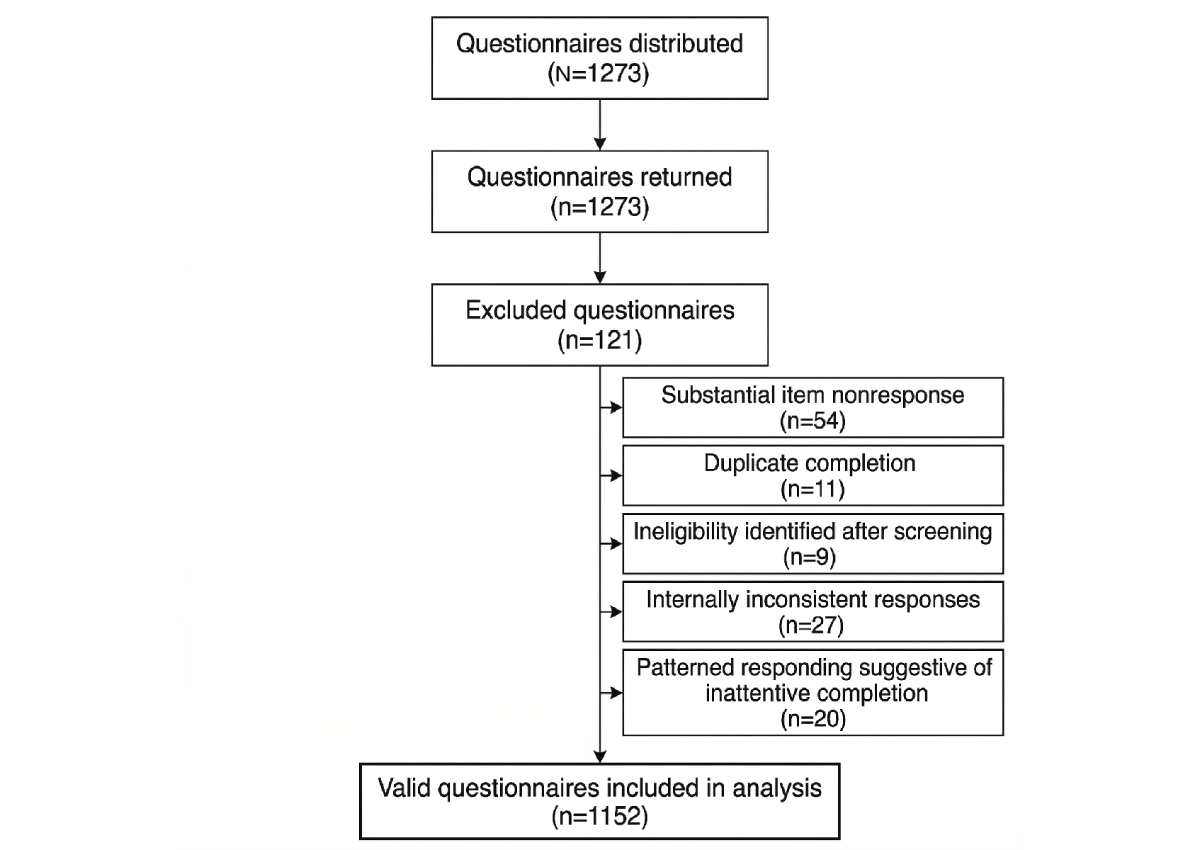
Participant recruitment and inclusion flowchart.

Participants completed the questionnaire in an average of 614 (range 472-922) seconds. Of 1273 questionnaires distributed, 1152 (90.5%) valid questionnaires were included in the final analysis. A total of 121 questionnaires were excluded because of substantial item nonresponse (n=54, 44.6%), duplicate completion (n=11, 9.1%), ineligibility identified after screening (n=9, 7.4%), internally inconsistent responses (n=27, 22.3%), or patterned responding suggestive of inattentive completion (n=20, 16.5%; Figure 1).

Among the 1152 participants, the mean age was 55.7 (SD 11.8) years. Overall, 52% (n=599) of the participants were female, 81.8% (n=942) resided in urban areas, 79.4% (n=915) were married, and 82.7% (n=953) had solid tumors. mHealth use was reported by 31.6% (n=364) of the participants, whereas 68.4% (n=788) reported no prior use. Variable-level missingness across the analyzed variables was low, and complete data on the outcome and all covariates required for the multivariable model were available for 97% (n=1118) of the participants.

As shown in Table 1, mHealth users were younger on average than nonusers (mean 52.4, SD 11.1 years vs mean 57.2, SD 11.9 years; *P*<.001) and were more likely to have undergraduate or postgraduate education (174/364, 47.8% vs 249/788, 31.6%; *P*<.001), an annual household income of at least ¥100,000 (US $14,527.90; 71/364, 19.5% vs 77/788, 9.8%; *P*<.001), and widowed or divorced marital status (72/364, 19.8% vs 100/788, 12.7%; *P*=.002) and to live alone (59/364, 16.2% vs 88/788, 11.2%; *P*=.02). Treatment dissatisfaction was also more common among users than nonusers (79/364, 21.7% vs 67/788, 8.5%; *P*<.001). No statistically significant group differences were observed for sex, residence, employment status, smoking, alcohol consumption, cancer type, or time since diagnosis.

**Table 1:** Participant characteristics overall and by mobile health (mHealth) use status (N=1152)^a^.

Characteristic			Total sample		mHealth users (n=364)		Nonusers (n=788)		*P* value	
Age (y), mean (SD)			55.7 (11.8)		52.4 (11.1)		57.2 (11.9)		<.001	
Female sex, n (%)			599 (52)		197 (54.1)		402 (51)		.33	
Urban residence, n (%)			942 (81.8)		309 (84.9)		633 (80.3)		.07	
**Educational level, n (%)**										<.001
	High school or lower	729 (63.3)		190 (52.2)		539 (68.4)				
	Undergraduate	357 (31)		138 (37.9)		219 (27.8)				
	Postgraduate or higher	66 (5.7)		36 (9.9)		30 (3.8)				
**Annual household income, n (%)**										<.001
	<¥50,000 (US $7263.95)	621 (53.9)		164 (45.1)		457 (58)				
	¥50,000 to ¥99,999 (US $7263.95 to US $14,527.80)	383 (33.2)		129 (35.4)		254 (32.2)				
	≥¥100,000 (US $14,527.90)	148 (12.8)		71 (19.5)		77 (9.8)				
**Marital status, n (%)**										.002
	Married	915 (79.4)		272 (74.7)		643 (81.6)				
	Unmarried	65 (5.6)		20 (5.5)		45 (5.7)				
	Widowed or divorced	172 (14.9)		72 (19.8)		100 (12.7)				
Living with others, n (%)			1005 (87.2)		305 (83.8)		700 (88.8)		.02	
Currently employed, n (%)			243 (21.1)		85 (23.4)		158 (20.1)		.18	
Current or former smoker, n (%)			182 (15.8)		63 (17.3)		119 (15.1)		.31	
Current alcohol use, n (%)			201 (17.4)		73 (20.1)		128 (16.2)		.10	
Solid tumor, n (%)			953 (82.7)		300 (82.4)		653 (82.9)		.84	
Time since diagnosis of ≥12 mo, n (%)			676 (58.7)		222 (61)		454 (57.6)		.29	
**Treatment satisfaction, n (%)**										<.001
	Satisfied	472 (41)		120 (33)		352 (44.7)				
	Neutral	534 (46.4)		165 (45.3)		369 (46.8)				
	Dissatisfied	146 (12.7)		79 (21.7)		67 (8.5)				

^a^*P* values were derived from independent-sample *t* tests for continuous variables and chi-square tests for categorical variables.

### Perceived Needs and Concerns Regarding mHealth Services Among All Participants

Across the full sample, perceived need for mHealth support was high. The most frequently endorsed needs were clinical guidance (917/1152, 79.6%), direct communication with health care professionals (901/1152, 78.2%), lifestyle guidance (846/1152, 73.4%), regular follow-up and evaluation (801/1152, 69.5%), and home-based disease management (780/1152, 67.7%). More than two-thirds of the participants (774/1152, 67.2%) also reported a need for access to reliable health information, whereas approximately half endorsed needs related to family assistance in disease management (628/1152, 54.5%), electronic medical record access (594/1152, 51.6%), peer support (562/1152, 48.8%), and online medication or equipment ordering (514/1152, 44.6%).

Concerns about mHealth services were also common. The most frequently reported concerns were leakage of private information (694/1152, 60.2%), inaccurate illness judgment (633/1152, 54.9%), and inaccurate information collection (596/1152, 51.7%). Reimbursement restrictions were endorsed by 42.9% (494/1152) of the participants, and 37.5% (432/1152) expressed concern about fraudulent or inauthentic physician information. Difficulty purchasing medicines through the platform was reported by 36.3% (418/1152) of the participants, whereas high service costs were reported by 27% (311/1152). Detailed frequencies are presented in Table 2.

**Table 2:** Perceived needs and concerns regarding mobile health services among all participants (N=1152).

Variable			Participants, n (%)
**Perceived needs^a^**			
	Clinical guidance	917 (79.6)	
	Direct communication with health care professionals	901 (78.2)	
	Lifestyle guidance	846 (73.4)	
	Regular follow-up and evaluation	801 (69.5)	
	Home-based disease management	780 (67.7)	
	Access to reliable health information	774 (67.2)	
	Family assistance in disease management	628 (54.5)	
	Electronic medical record access	594 (51.6)	
	Peer support	562 (48.8)	
	Online medication or equipment ordering	514 (44.6)	
**Concerns and barriers^a^**			
	Leakage of private information	694 (60.2)	
	Inaccurate illness judgment	633 (54.9)	
	Inaccurate information collection	596 (51.7)	
	Reimbursement restrictions	494 (42.9)	
	Fraudulent or inauthentic physician information	432 (37.5)	
	Difficulty purchasing medicines through the platform	418 (36.3)	
	High service costs	311 (27)	

^a^Multiple responses were permitted; percentages do not add up to 100%.

### Factors Associated With mHealth Use

Variables significantly associated with mHealth use in univariate analyses were entered into the multivariable logistic regression model. The adjusted model was based on 1118 participants with complete data on the outcome and all included covariates. In the final model, younger age, higher educational level, higher annual household income, widowed or divorced marital status, living alone, and treatment dissatisfaction were independently associated with higher odds of mHealth use. The model showed acceptable fit (Hosmer-Lemeshow *P*=.60) and moderate discrimination (area under the receiver operating characteristic curve=0.74).

**Figure 2:**
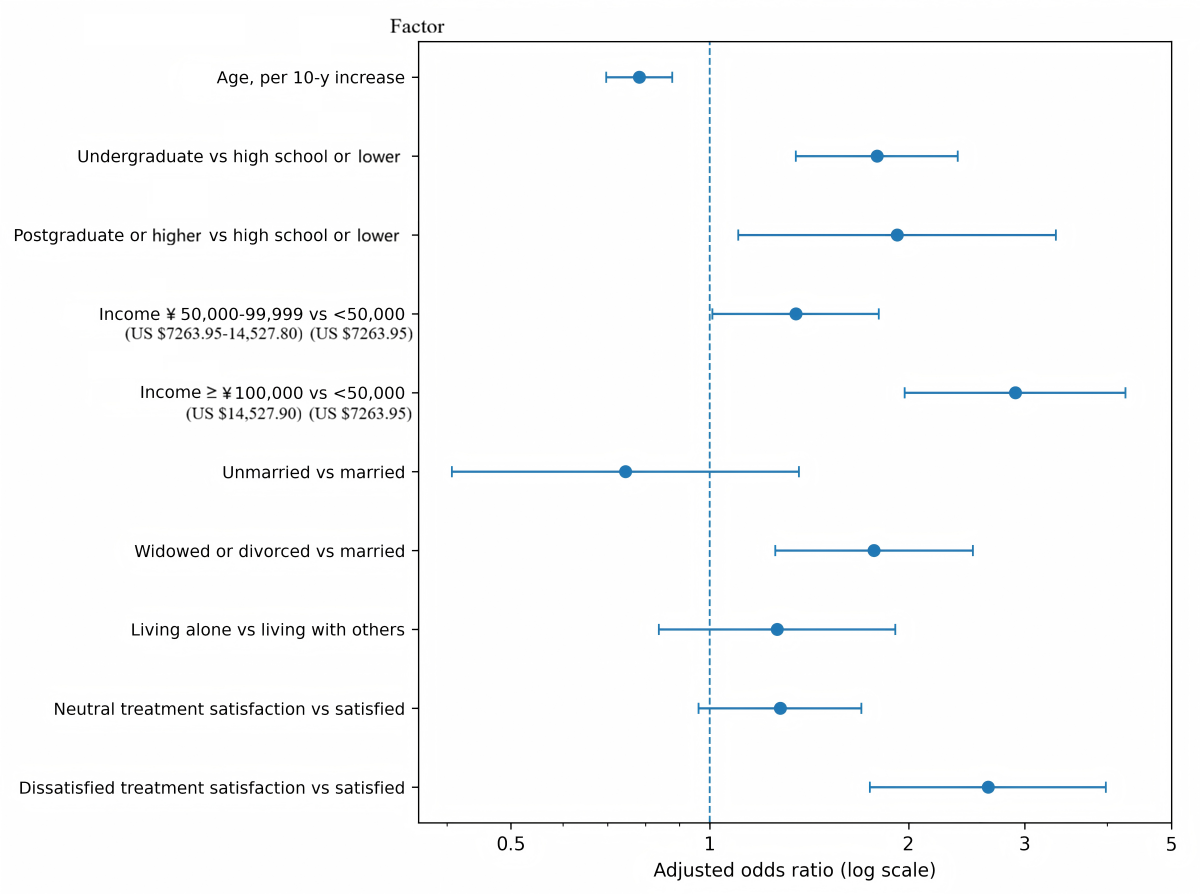
Adjusted odds ratios for factors associated with mobile health (mHealth) use. Forest plot of adjusted odds ratios from the multivariable logistic regression model computed from the dataset. Younger age, higher educational level, higher annual household income, widowed or divorced marital status, and treatment dissatisfaction were associated with greater odds of mHealth use. Error bars represent 95% CIs.

In the adjusted model, the strongest positive associations with mHealth use were observed for postgraduate educational level, treatment dissatisfaction, and annual household income of at least ¥100,000 (US $14,527.90). Undergraduate educational level, widowed or divorced marital status, and living alone were also significantly associated with higher odds of use, whereas older age was significantly associated with lower odds of use (*P*≤.046 in all cases). In contrast, annual household income of ¥50,000 (US $7263.95) to ¥99,999 (US $14,527.80), unmarried status, and neutral treatment satisfaction were not statistically significant (*P*≥.10 in all cases; Table 3; Figure 2).

**Table 3:** Multivariable logistic regression analysis of factors associated with mobile health use (n=1118).

Variable		Adjusted OR^a^ (95% CI)	*P* value
Age per 10-y increase		0.82 (0.73-0.92)	<.001
**Educational level**			
	High school or lower	Reference	—^b^
	Undergraduate	1.73 (1.29-2.33)	<.001
	Postgraduate or higher	3.12 (1.82-5.34)	<.001
**Annual household income**			
	<¥50,000 (US $7263.95)	Reference	—
	¥50,000 to ¥99,999 (US $7263.95 to US $14,527.80)	1.24 (0.92-1.67)	.16
	≥¥100,000 (US $14,527.90)	2.21 (1.45-3.37)	<.001
**Marital status**			
	Married	Reference	—
	Unmarried	1.02 (0.56-1.84)	.95
	Widowed or divorced	1.68 (1.17-2.42)	.005
Living alone		1.49 (1.01-2.21)	.046
**Treatment satisfaction**			
	Satisfied	Reference	—
	Neutral	1.28 (0.95-1.72)	.10
	Dissatisfied	3.08 (2.02-4.68)	<.001

^a^OR: odds ratio.

^b^Not applicable.

### Patterns of mHealth Use Among Users

Among the 364 mHealth users, the most commonly reported frequency of use was monthly (n=126, 34.6%), followed by weekly (n=118, 32.4%), less than monthly (n=62, 17%), and daily (n=58, 15.9%). The mean duration of a typical use session was 22.4 (SD 10.2) minutes. Most users (n=244, 67%) primarily accessed services through WeChat-based platforms, whereas 25% (n=91) primarily used dedicated mHealth apps and 8% (n=29) primarily used hospital web-based portals or hospital apps.

Appointment booking (301/364, 82.7%) and online consultation (244/364, 67%) were the most commonly used functions, followed by viewing examination or laboratory reports (215/364, 59.1%), accessing health information (185/364, 50.8%), medication reminders (128/364, 35.2%), and symptom self-monitoring (117/364, 32.1%). Peer support or patient community functions were less commonly used (69/364, 19%). Overall satisfaction with mHealth services was favorable, with 67.3% (245/364) of the users reporting that they were satisfied or very satisfied with their experience (Table 4).

**Table 4:** Patterns of mobile health (mHealth) use among users (n=364).

Variable			Participants, n (%)
**Frequency of use**			
	Daily	58 (15.9)	
	Weekly	118 (32.4)	
	Monthly	126 (34.6)	
	Less than monthly	62 (17)	
Typical session duration (min), mean (SD)			22.4 (10.2)
**Primary platform used**			
	WeChat-based platform	244 (67)	
	Dedicated mHealth app	91 (25)	
	Hospital website or hospital app	29 (8)	
**Commonly used functions^a^**			
	Appointment booking	301 (82.7)	
	Online consultation	244 (67)	
	Viewing examination or laboratory reports	215 (59.1)	
	Accessing health information	185 (50.8)	
	Medication reminders	128 (35.2)	
	Symptom self-monitoring	117 (32.1)	
	Peer support or community interaction	69 (19)	
**Overall satisfaction with mHealth services**			
	Very satisfied	61 (16.8)	
	Satisfied	184 (50.5)	
	Neutral	93 (25.5)	
	Dissatisfied	26 (7.1)	

^a^Multiple responses were permitted for commonly used functions; percentages for that section do not add up to 100%.

### User Experience and System Performance Among mHealth Users

User-reported experience with mHealth services was generally positive but not uniformly so. Among users, 66.2% (241/364) described the services as easy or very easy to use, whereas 8.5% (31/364) described them as difficult. In terms of convenience, 68.4% (249/364) of the users rated the services as somewhat or very convenient, whereas 7.1% (26/364) rated them as inconvenient. System stability was moderate: 38.7% (141/364) of the users reported never experiencing crashes, 37.6% (137/364) reported crashes rarely, 19.2% (70/364) reported them sometimes, and 4.4% (16/364) reported them often.

Perceptions of clinical utility were more mixed. Only 40.9% (149/364) of users believed that the mHealth services they used could provide disease-specific treatment support, whereas 40.7% (148/364) believed that they could not and 18.4% (67/364) were uncertain. Perceived integration with hospital services was more favorable, with 61.3% (223/364) of users reporting that the service they used was integrated with hospital-based care, 17.6% (64/364) being uncertain, and 21.2% (77/364) reporting no integration. Additional user experience indicators are presented in Table 5.

**Table 5:** User experience and system performance among mobile health users (n=364).

Variable		Participants, n (%)
**Ease of use**		
	Very easy	76 (20.9)
	Easy	165 (45.3)
	Neutral	92 (25.3)
	Difficult	31 (8.5)
**Overall convenience**		
	Very convenient	68 (18.7)
	Somewhat convenient	181 (49.7)
	Neutral	89 (24.5)
	Inconvenient	26 (7.1)
**Frequency of system crashes**		
	Never	141 (38.7)
	Rarely	137 (37.6)
	Sometimes	70 (19.2)
	Often	16 (4.4)
**Advertising burden**		
	Few advertisements	158 (43.4)
	Moderate number of advertisements	112 (30.8)
	Many advertisements	94 (25.8)
**Can provide disease-specific treatment support**		
	Yes	149 (40.9)
	Uncertain	67 (18.4)
	No	148 (40.7)
**Integrated with hospital services**		
	Yes	223 (61.3)
	Uncertain	64 (17.6)
	No	77 (21.2)

## Discussion

### Principal Findings

In this multicenter cross-sectional survey study of adult survivors of cancer in China, approximately one-third of participants (364/1152, 31.6%) reported prior mHealth use. Use was concentrated on practical care navigation functions, especially appointment booking, online consultation, and report viewing, and most users (244/364, 67%) accessed services through WeChat-based platforms rather than stand-alone apps. In adjusted analyses, mHealth use was associated primarily with age, educational level, income, social circumstances, and treatment dissatisfaction rather than with clinical characteristics. At the same time, survivors expressed a strong demand for clinically relevant functions, especially guidance and direct communication with health care professionals, while also reporting substantial concerns about privacy, accuracy, reimbursement, and physician authenticity. Taken together, the findings suggest that mHealth is already a meaningful component of survivorship care for some patients, but its use remains selective rather than broad based. Current engagement appears to be driven more by immediate practical needs and perceived gaps in care than by mature adoption of comprehensive digital survivorship support.

### Comparison With Prior Work and Interpretation of Correlates

The adjusted associations observed in this study suggest that mHealth use among adult survivors of cancer in China is shaped less by clinical characteristics than by digital readiness and perceived unmet needs. Younger age, higher educational level, and higher income were associated with greater mHealth use, a pattern that is consistent with that observed in prior literature showing that digital health uptake is socially patterned and facilitated by greater digital access, confidence, and ability to navigate app-based care [28,29]. In contrast, cancer type and time since diagnosis were not significant in the adjusted model, suggesting that engagement with mHealth in this sample was not primarily determined by the disease profile itself [30].

The social and care experience correlates point in a complementary direction. Widowed or divorced marital status, living alone, and treatment dissatisfaction were associated with higher mHealth use, which may reflect greater motivation to seek supplementary support when day-to-day support is more limited or when conventional care is perceived as insufficient [24,31]. Viewed together, these findings suggest that the significant correlates are connected rather than coincidental: some factors may increase capacity to engage with mHealth, whereas others may increase motivation to use it [28,31]. This interpretation helps explain why uptake appears to be socially and experientially patterned rather than primarily disease driven, and it suggests that implementation strategies should not rely on clinical stratification alone because real-world use may depend more on who is able and motivated to engage with mHealth [24,32].

### Perceived Needs for mHealth Services

The needs profile observed in this study suggests that survivors are seeking clinically meaningful support rather than purely informational content [33,34]. The highest-demand functions centered on guidance, communication, follow-up, and home-based disease management, indicating that survivors view mHealth as a potential extension of survivorship care rather than merely a source of general health information [33,34].

The prominence of communication-related needs is especially important. The finding that 78.2% (901/1152) of the participants desired direct communication with health care professionals suggests that digital health tools may be most acceptable when they enhance continuity and responsiveness rather than when they merely provide static educational content [33,34]. Similarly, the high demand for clinical guidance and follow-up indicates that survivors may value digital services most when they support ongoing management rather than one-time information retrieval [33]. This interpretation is reinforced by the use pattern findings, where appointment booking, online consultation, and result viewing were much more common than peer support functions. Together, these results suggest that survivors prioritize utility, accessibility, and clinical relevance over more discretionary or socially oriented features [33].

The demand for electronic medical record access, reliable health information, and family assistance in disease management also points to the importance of integrating mHealth into broader care systems [14,35,36]. Survivors are not simply asking for more digital tools; they appear to be asking for tools that help them coordinate care, interpret medical information, and manage illness in the home environment [24,37]. This has implications for future design. mHealth interventions for cancer survivorship are likely to be more valuable when they are linked to clinical workflows, adapted to family involvement, and structured around specific self-management tasks rather than around generic health promotion messages [17,36,38].

### Concerns, Barriers, and User Experience

Although the perceived need for mHealth services was high, concerns and barriers were also prominent. The most common concerns centered on privacy, accuracy, and reliability, indicating that survivors are attentive not only to what digital services can offer but also to the risks associated with using them [39,40]. Privacy concerns are particularly important in oncology settings, where personal health information may be perceived as sensitive and stigma may remain relevant in some contexts [41]. Concerns about inaccurate judgment and inaccurate information collection point to a deeper issue of clinical trust [42,43]. Survivors may be willing to use digital services for administrative functions while remaining uncertain about their reliability for individualized assessment or treatment-related decision support [39,44,45].

The concern about reimbursement restrictions, reported by 42.9% (494/1152) of the participants, should also be taken seriously. This is not a marginal finding. Rather, it suggests that payment arrangements and insurance integration remain practical barriers to wider adoption [24,40,44]. Even well-designed digital services may remain underused if they are not financially accessible or if their status within formal reimbursement structures is unclear [39,40]. Similarly, more than one-third of participants (432/1152, 37.5%) expressed concern about fraudulent or inauthentic physician information, which highlights the role of physician verification and institutional credibility. In this context, trust is not only a technical issue but also a governance issue [35,39].

The user experience findings suggest that current mHealth services are generally usable and convenient but are not yet consistently trusted as tools for disease-specific clinical support. Therefore, improving usability alone is unlikely to be sufficient; stronger clinical linkage, transparent quality assurance, and clearer integration with formal oncology care may be necessary to improve trust and sustain engagement [39,42,45].

### Implications for Practice and Research

These findings have several practical implications. Survivorship-oriented mHealth services should prioritize functions that survivors clearly value, particularly clinical guidance, communication, follow-up, and home-based disease management. Because uptake appears to be facilitated by familiarity and convenience, integration into commonly used platforms such as WeChat may be more effective than reliance on stand-alone apps alone. At the same time, the social patterning of use suggests that implementation should include explicit support for older adults and survivors with fewer educational or financial resources.

Trust building should also be central to implementation. Privacy protection, physician authentication, content quality control, and clearer reimbursement pathways may influence uptake as much as interface design. Future research should examine patterns of sustained use over time, discontinuation, and whether mHealth engagement improves survivorship outcomes in specific patient subgroups.

### Strengths and Limitations

This study has several strengths. It used a multicenter design, included a relatively large sample of adult survivors of cancer, and achieved a high effective response rate. It also examined not only whether survivors used mHealth services but also how they used them, what they wanted from them, and what concerns they had. This broader perspective is valuable because uptake alone provides an incomplete picture of digital health implementation in survivorship care.

Several limitations should also be considered. First, the cross-sectional design precludes causal inference, and the observed associations should not be interpreted as directional. For example, treatment dissatisfaction may increase mHealth use, but mHealth use may also shape how survivors evaluate their care experiences. Second, participants were recruited using convenience sampling from tertiary cancer centers, which may limit generalizability to survivors receiving care in other settings, particularly rural or lower-resource environments. Although the effective response rate was high, some degree of self-selection bias likely remains because participation depended on survivors being present, eligible, and willing to complete the survey. Third, all variables were self-reported and, therefore, are subject to recall bias and response bias. This is especially relevant for prior mHealth use, perceived needs, treatment satisfaction, and user experience items. Fourth, missing data were limited, and the Little MCAR test supported the use of complete-case analysis, but this approach may still have reduced precision and could introduce bias if the true missing data mechanism deviated from MCAR. Finally, although the questionnaire was developed from prior literature, reviewed by experts, piloted, and showed acceptable internal consistency for several multi-item domains, it was not a fully validated instrument. Accordingly, some measurement error or limitations in construct validity may remain.

### Conclusions

mHealth use among adult survivors of cancer in China is established but uneven, with use concentrated on practical functions such as appointment booking, online consultation, and result viewing rather than comprehensive survivorship support. Uptake was significantly associated with younger age, higher educational level, higher income, widowed or divorced marital status, and treatment dissatisfaction but not with clinical characteristics, suggesting that digital engagement reflects both resource-related differences and perceived unmet needs. Survivors reported a strong demand for clinically relevant functions, especially guidance, communication, follow-up, and home-based management, but also expressed substantial concerns about privacy, accuracy, reimbursement, and physician authenticity. These findings indicate that future survivorship mHealth services should prioritize clinical relevance, trust, integration with formal care, and equitable implementation if they are to achieve broader and more meaningful use.

## Data Availability

The datasets generated or analyzed during this study are available from the corresponding author on reasonable request.

## Appendix

Multimedia Appendix 1 Questionnaire (English translation).

## Abbreviations

MCAR: missing completely at random
mHealth: mobile health
QOL: quality of life
