# Supplementary material for: Use and Influencing Factors of mHealth Services Among Adult Survivors of Cancer: Cross-Sectional Survey Study
Source: J Med Internet Res. 2026 Apr 23;28:e82902. doi: 10.2196/82902 (PMC13153749; doi:10.2196/82902)
Supplement: Multimedia Appendix 1 [file jmir_v28i1e82902_app1.pdf]

Appendix 1. Questionnaire (English Translation)

Survey Title

Questionnaire on Utilization and Influencing Factors of mHealth Services Among Adult Cancer Survivors in China

Screen 1. Information and Electronic Consent (required)

You are invited to take part in a research survey about the use of mobile health services among people diagnosed with cancer. The survey is anonymous and does not ask for your name or ID number. Participation is voluntary. Refusal will not affect your medical care. You may stop at any time by closing the survey. Please be advised that some questions may feel sensitive. Your answers will be used only for research and reported in summary form. If you have questions, ask the on-site researcher.

Q0. Do you agree to participate in this survey? (single choice, required)
1 Yes, I agree and want to continue
2 No, I do not agree (end survey)

Screen 2. Eligibility (required)
Q1. Are you 18 years old or older? (single choice, required)
1 Yes
2 No (end survey)

Q2. Have you ever been diagnosed with cancer (malignant tumor) by a doctor? (single choice, required)
1 Yes
2 No (end survey)

Q3. Are you able to complete this questionnaire on a smartphone by yourself (with reading assistance if needed)? (single choice, required)
1 Yes
2 No (end survey)

Screen 3. Sociodemographic Characteristics
Q4. Sex (single choice, required)
1 Male
2 Female

Q5. Age (years) (numeric entry, required)
Please enter your age in full years: ___

Q6. Highest education level completed (single choice, required)
1 High school or below
2 Undergraduate (college/university)

3 Postgraduate or above

Q7. Average annual household income (single choice, required)
1 < ¥50,000
2 ¥50,000–¥100,000
3 ≥ ¥100,000

Q8. Marital status (single choice, required)
1 Married
2 Unmarried (never married)
3 Widowed or divorced

Q9. Current residence (single choice, required)
1 Urban area
2 Rural area

Q10. Employment status (single choice, required)
1 Employed
2 Unemployed (includes retired, homemaker, unable to work)

Q11. Do you currently live with other people? (single choice, required)
1 Yes (living with others)
2 No (living alone)

Q12. Smoking status (single choice, required)
1 Current smoker
2 Former smoker
3 Never smoker

Q13. Alcohol consumption (single choice, required)
1 Drank alcohol within the past month
2 Drank alcohol before but not within the past month
3 Never drank alcohol

Screen 4. Clinical Characteristics
Q14. Cancer type (single choice, required)
Instruction: Solid tumors include cancers such as lung, breast, colorectal, liver, stomach, thyroid, and others. Non-solid tumors include blood cancers such as leukemia, lymphoma, and myeloma.
1 Solid tumor
2 Non-solid tumor

Q15. Time since first cancer diagnosis (single choice, required)
1 Less than 12 months
2 12 months or more

Q16. Satisfaction with cancer treatment received so far (single choice, required)
1 Satisfied
2 Neutral
3 Dissatisfied

Screen 5. mHealth Adoption (primary outcome)
Definition: In this survey, "mHealth services" refer to patient-facing health-related services delivered via mobile phones and apps or platforms, including hospital-affiliated WeChat official accounts or mini-programs and other medical apps that can be used for functions such as appointment booking and online consultation.

Q17. Have you ever used any mHealth services for your health or cancer-related care? (single choice, required)
1 Yes
2 No
Skip logic: If "No," skip to Screen 10; if "Yes," continue to Screen 6.

Screen 6. Patterns of mHealth Use (users only)
Q18. How often do you use mHealth services? (single choice, required)
1 Daily
2 Weekly
3 Monthly
4 Occasionally (< monthly)

Q19. On average, how long do you spend per use/session? (single choice, required)
1 < 5 min
2 5–10 min
3 11–20 min
4 21–30 min
5 > 30 min

Q20. Which platforms do you mainly use for mHealth services? (single choice, required)
1 WeChat health services (hospital-affiliated official account or mini-program)
2 Specialized medical/health apps (non-WeChat)

Q21. Which functions have you used in mHealth services? (multiple choice, required)
1 Appointment booking
2 Online consultation
3 Health information retrieval
4 Medication reminders

Q22. Overall satisfaction with mHealth services (single choice, required)
1 Very satisfied

2 Satisfied
3 Neutral
4 Dissatisfied
5 Very dissatisfied
Analysis note: combine 1+2 = “satisfied/very satisfied”; 4+5 can be merged with 3 or excluded.

Screen 7. Needs and Expectations for mHealth Functions (users only)
Instruction: Please indicate how much you would want each function in mHealth services for cancer survivorship care.
Response scale (single choice each): 1 Strongly need2 Somewhat need3 Neutral4 Somewhat do not need5 Do not need at all

Q23 Clinical guidance (symptom advice, treatment-related guidance)
1 Strongly need
2 Somewhat need
3 Neutral
4 Somewhat do not need
5 Do not need at all

Q24 Communication with health-care providers
1 Strongly need
2 Somewhat need
3 Neutral
4 Somewhat do not need
5 Do not need at all

Q25 Lifestyle guidance (diet, exercise, sleep)
1 Strongly need
2 Somewhat need
3 Neutral
4 Somewhat do not need
5 Do not need at all

Q26 Home-based disease management
1 Strongly need
2 Somewhat need
3 Neutral
4 Somewhat do not need
5 Do not need at all

Q27 Access to health information/health education
1 Strongly need
2 Somewhat need
3 Neutral

4 Somewhat do not need
5 Do not need at all

Q28 Regular follow-up and evaluation/reminders
1 Strongly need
2 Somewhat need
3 Neutral
4 Somewhat do not need
5 Do not need at all

Q29 Family assistance in management
1 Strongly need
2 Somewhat need
3 Neutral
4 Somewhat do not need
5 Do not need at all

Q30 Peer support
1 Strongly need
2 Somewhat need
3 Neutral
4 Somewhat do not need
5 Do not need at all

Q31 Access to electronic medical records
1 Strongly need
2 Somewhat need
3 Neutral
4 Somewhat do not need
5 Do not need at all

Q32 Online drug or rehabilitation equipment ordering
1 Strongly need
2 Somewhat need
3 Neutral
4 Somewhat do not need
5 Do not need at all
Analysis: define “demand” as options 1–2.

Screen 8. Concerns and Barriers (users only)
Instruction: Please indicate how worried you are about each potential problem when using mHealth services.
Response scale (single choice each): 1 Very worried2 Somewhat worried3 Not very worried4 Not worried at all

Q33 Privacy leakage
1 Very worried
2 Somewhat worried
3 Not very worried
4 Not worried at all

Q34 Inaccurate information collection
1 Very worried
2 Somewhat worried
3 Not very worried
4 Not worried at all

Q35 Inaccurate illness judgment
1 Very worried
2 Somewhat worried
3 Not very worried
4 Not worried at all

Q36 Reimbursement restrictions
1 Very worried
2 Somewhat worried
3 Not very worried
4 Not worried at all

Q37 Inauthentic physician information
1 Very worried
2 Somewhat worried
3 Not very worried
4 Not worried at all

Q38 Limitations in purchasing medicines
1 Very worried
2 Somewhat worried
3 Not very worried
4 Not worried at all

Q39 High service costs
1 Very worried
2 Somewhat worried
3 Not very worried
4 Not worried at all
Analysis: combine 1+2 = “very/somewhat worried”.

Screen 9. User-Experience Issues (users only)

Q40 Convenience of mHealth services (single choice, required)
1 Very convenient
2 Somewhat convenient
3 Neutral
4 Not convenient

Q41 System crashes (single choice, required)
1 Frequent
2 Occasional
3 Rare
4 Never

Q42 Advertisements in mHealth services (single choice, required)
1 Many
2 Moderate
3 Few
4 None

Q43 Concern about fraud or scams (single choice, required)
1 Worried (very or somewhat)
2 Not worried

Q44 Ease of operation (single choice, required)
1 Easy or very easy
2 Neutral
3 Difficult

Q45 Ability of mHealth services to deliver disease-specific treatment support (single choice, required)
1 Yes
2 No
3 Uncertain

Q46 Integration with hospital information systems (single choice, required)
1 Integrated
2 Not integrated
3 Uncertain

Screen 10. Optional Open-Ended Feedback (all participants)

Q47 Do you have any other comments or suggestions about using mHealth services for cancer care? (open text, optional)
[Free-text box]
